# Issues in characterizing resting energy expenditure in obesity and after weight loss

**DOI:** 10.3389/fphys.2013.00047

**Published:** 2013-03-25

**Authors:** Anja Bosy-Westphal, Wiebke Braun, Britta Schautz, Manfred J. Müller

**Affiliations:** ^1^Institut für Humanernährung und Lebensmittelkunde, Christian-Albrechts-Universität zu KielKiel, Germany; ^2^Institut für Ernährungsmedizin, Universität HohenheimStuttgart, Germany

**Keywords:** resting energy expenditure, normalization, fat free mass, fat mass, organ mass, obesity weight loss, adaptive thermogenesis

## Abstract

**Limitations of current methods:** Normalization of resting energy expenditure (REE) for body composition using the 2-compartment model fat mass (FM), and fat-free mass (FFM) has inherent limitations for the interpretation of REE and may lead to erroneous conclusions when comparing people with a wide range of adiposity as well as before and after substantial weight loss.

**Experimental objectives:** We compared different methods of REE normalization: (1) for FFM and FM (2) by the inclusion of %FM as a measure of adiposity and (3) based on organ and tissue masses. Results were compared between healthy subjects with different degrees of adiposity as well as within subject before and after weight loss.

**Results:** Normalizing REE from an “REE vs. FFM and FM equation” that (1) was derived in obese participants and applied to lean people or (2) was derived before weight loss and applied after weight loss leads to the erroneous conclusion of a lower metabolic rate (i) in lean persons and (ii) after weight loss. This is revealed by the normalization of REE for organ and tissue masses that was not significantly different between lean and obese or between baseline and after weight loss. There is evidence for an increasing specific metabolic rate of FFM with increasing %FM that could be explained by a higher contribution of liver, kidney and heart mass to FFM in obesity. Using “REE vs. FFM and FM equations” specific for different levels of adiposity (%FM) eliminated differences in REE before and after weight loss in women.

**Conclusion:** The most established method for normalization of REE based on FFM and FM may lead to spurious conclusions about metabolic rate in obesity and the phenomenon of weight loss-associated adaptive thermogenesis. Using %FM-specific REE prediction from FFM and FM in kg may improve the normalization of REE when subjects with wide differences in %FM are investigated.

## Introduction

In order to differentiate phenotypes of high and low metabolic rate or to understand the changes in resting energy expenditure (REE) that accompany weight loss or gain normalization of REE is required (Arch et al., 2006; Kaiyala and Schwartz, 2011). Larger people naturally have a higher REE than smaller people. However, using body mass as a parameter for REE, normalization would lead to the errant conclusion that obese people have a lower REE than lean individuals. Because in obese people body mass consists of a higher proportion of metabolically inert fat mass (FM), the specific metabolic rate (i.e., energy expenditure per unit body mass) is lower. Body composition is therefore a crucial determinant of REE.

Owing to the widespread use of the 2-compartment model of body composition, normalization of REE is generally performed by accounting for metabolically active fat-free mass (FFM). FFM is the main determinant of REE explaining between 53 and 88% (usually not more than 75%) of its variance (reviewed by Nelson et al., 1992). REE scales with FFM, the regression line has a significant intercept term of about 400 kcal/day (Müller et al., 2011; Heymsfield et al., 2012). Numerous regression equations have been published for prediction of REE from FFM (reviewed by Wang et al., 2000). These equations all have similar slopes varying from 19.7 to 24.5 kcal × kg FFM^−1^ × day^−1^ and positive intercepts varying from 186 to 662 kcal/day (Wang et al., 2000). Differences in the methodology used to estimate FFM did not explain the discrepancies between those equations that may rather be due to population differences (Korth et al., 2007).

The volume and mass of individual tissues can now be measured with great accuracy and a new reference man has been proposed (Later et al., 2010). Reference data on detailed body composition of young normal weight adults are given in Table 1. Very recently these data have been used to explain the non-zero intercept of the REE-FFM function (Heymsfield et al., 2012). Using different models including adipose tissue free mass, adipose tissue, sex as well as individual organ masses the explained variance was increased. When compared with the other organs, brain relates differently to FFM (i.e., in adults, brain mass does not change much with body weight); therefore, in the last model brain mass was added which reduced the intercept from 410 to non-significant, i.e., 54 kcal/day (Heymsfield et al., 2012).

**Table 1:** Detailed body composition in a healthy adult reference population^*^.

	**Men**		**Women**		***P*-value**
	**Mean ± *SD***	**Range**	**Mean ± *SD***	**Range**	
Age, years	31.6 ± 5.8	20−40	30.7 ± 5.9	20−40	0.344
Height, m	1.80 ± 0.06	1.68−1.93	1.69 ± 0.07	1.49−1.86	<0.001
Weight, kg	90.2 ± 18.9	58.2−137.1	85.3 ± 22.7	44.7−144.9	0.161
BMI, kg/m^2^	27.6 ± 5.2	19.0−41.9	29.9 ± 7.4	16.8−46.77	<0.05
WC, cm	95.4 ± 15.0	67.5−128.6	94.9 ± 17.6	65.0−131.0	0.862
HC, cm	103.1 ± 10.5	83.0−127.0	111.2 ± 16.3	77.5−153.0	<0.001
**FM**					
FM, %	23.1 ± 9.3	5.3−41.3	38.8 ± 10.9	11.0−57.9	<0.001
FM, kg	22.3 ± 12.8	3.1−54.7	35.2 ± 17.7	5.9−83.9	<0.001
FMI, kg/m^2^	6.8 ± 3.8	1.0−16.7	12.3 ± 6.0	1.9−26.3	<0.001
**AT, l**					
SAT total	20.6 ± 8.5	7.7−44.3	33.8 ± 15.1	11.0−75.9	<0.001
SAT arms	2.5 ± 0.9	0.3−5.4	3.4 ± 1.5	1.0−7.5	<0.001
SAT trunk	9.6 ± 4.9	2.6−22.1	15.9 ± 7.8	3.3−36.2	<0.001
SAT legs	8.5 ± 3.3	2.9−18.4	15.2 ± 6.7	5.2−34.9	<0.001
VAT	3.9 ± 2.3	0.6−9.3	2.0 ± 1.2	0.3−5.8	<0.001
BrAT	−	−	1.7 ± 0.9	0.4−4.4	
**TBW**					
TBW, l	51.2 ± 9.0	36.4−76.5	38.9 ± 7.3	25.6−56.8	<0.001
ECW, l	17.8 ± 2.4	13.2−25.7	15.3 ± 2.6	10.5−23.5	<0.001
ICW, l	30.0 ± 3.7	22.1−39.6	23.2 ± 5.5	14.2−37.0	<0.001
**SM, kg**					
SM total	34.1 ± 5.8	23.3−49.7	22.8 ± 4.2	14.4−34.8	<0.001
SM arms	5.0 ± 0.9	3.2−6.8	3.0 ± 0.6	1.8−4.6	<0.001
SM trunk	12.8 ± 2.5	8.1−19.3	8.1 ± 1.7	4.8−13.5	<0.001
SM legs	16.4 ± 3.3	11.1−24.4	11.7 ± 2.2	6.8−17.5	<0.001
**ORGAN AND BONE MASS, kg**					
Bone mass	5.4 ± 0.7	4.2−6.8	4.5 ± 0.7	3.0−7.3	<0.001
Brain	1.61 ± 0.12	1.34−1.96	1.45 ± 0.09	1.23−1.75	<0.001
Liver	1.81 ± 0.39	1.16−2.87	1.61 ± 0.32	0.94−2.59	<0.001
Heart	0.34 ± 0.08	0.22−0.57	0.28 ± 0.07	0.17−0.54	<0.001
Kidneys	0.32 ± 0.06	0.20−0.44	0.29 ± 0.08	0.16−0.54	<0.05
Spleen	0.33 ± 0.11	0.17−0.67	0.24 ± 0.10	0.09−0.56	<0.001
Residual	24.0 ± 3.6	16.2−33.7	18.7 ± 3.2	13.0−28.5	<0.001

Comparison between men (n = 58) and women (n = 117) between 20 and 40 years.

* A detailed description of the study protocol, inclusion criteria, subjects, and methods has been published previously (Later et al., 2010). BMI, body mass index; WC, waist circumference; HC, hip circumference; FM, fat mass; FMI, fat mass index; AT, adipose tissue; SAT, subcutaneous adipose tissue; VAT, visceral adipose tissue; BrAT, breast adipose tissue; TBW, total body water; ECW, extracellular water; ICW, intracellular water; SM, skeletal muscle mass.

Normalization of REE for FFM using regression analysis is a statistical rather than a physiological approach (Brozek and Grande, 1955; Garby et al., 1988). FFM is chemically defined as the mass of the body when ether-soluble material has been removed (body weight—FM). However, even though FFM does not differ much from the physiologic lean body mass which is the mass of all tissues in the body excluding adipose tissue (Garrow, 1974), FFM remains a heterogeneous compartment and the specific metabolic rate of FFM decreases with increasing FFM. This is explained by an increase in adipose tissue-derived FFM (water, protein, and minerals) with increasing body weight, but also by a concomitant decrease in the proportion of high metabolically active organ mass (brain, liver, kidney, and heart mass) compared to low metabolically active lean body mass (connective tissue, skeletal muscle mass, bone mass, Wang et al., 2000).

Besides FFM, FM explains a small additional proportion in REE variance, especially in populations with a wide range of adiposity (Nelson et al., 1992). The contribution of FM appears to be around 0.15–0.2 of the contribution of the same mass of FFM (Garrow and Webster, 1985; Ravussin et al., 1986; Nelson et al., 1992) or even a greater proportion of that of the equivalent FFM in patients with type 2 diabetes (Bitz et al., 2004). Physiological interpretation of the regression coefficient for FM is difficult and can only partly be accomplished by the low metabolic rate of adipose tissue (i.e., 11.3–14.3 kJ × kg lipid^−1^ × 24 h^−1^, Hallgren et al., 1989; Müller et al., 2009). In mice, the contribution of FM to the variance in REE is substantially greater than predicted from the metabolic cost of adipose tissue *per se* (Kaiyala et al., 2010). The mechanism underlying this effect may be an indirect impact of FM on REE (Bosy-Westphal et al., 2009). In this regard, gains and losses in FM are assumed to trigger compensatory adjustments in REE (e.g., an adipose-tissue specific control of thermogenesis, Dulloo and Jacquet, 1998) and adipose tissue may be coupled to energy homeostasis by “adiposity signals” like leptin and insulin that act on the central nervous system and affect autonomic and behavioral outputs that are directed toward a restoration of energy or fat balance (Morton et al., 2006). These factors might contribute to an increase in REE in obesity and might partly explain how FM can be a major determinant of REE although adipose tissue itself only has a low metabolic rate (Kaiyala et al., 2010).

However, the contribution of FM to metabolic rate in humans largely remains causally enigmatic. The complexity of this contribution is illustrated by our previous results which show that the regression coefficients for FFM and FM differ between different degrees of adiposity (Bosy-Westphal et al., 2009) thus inevitably leading to a yet unsolved problem of REE normalization in different degrees of adiposity when using the same regression equation. Insufficient normalization of energy expenditure also led to the misinterpretation of a low metabolic rate in ob/ob mice (see Butler and Kozak, 2010; Tschöp et al., 2011 for review).

The aim of this contribution is to show that the most established method for normalization of REE based on FFM and FM may lead to spurious conclusions about the phenomenon of weight loss-associated adaptive thermogenesis (i.e., a reduction of REE beyond what is explained by a change in body composition). Adaptive thermogenesis, in obese people who have lost weight, may be overestimated or even seen as an artefact explained by the inadequate normalization for FM. Likewise, we hypothesize that the common procedure of REE normalization for FFM and FM becomes awkward when the metabolic rates of lean and obese persons are compared. In order to test our hypotheses, we compared different methods of REE normalization (1) for FFM and FM, (2) including %FM as a measure of adiposity and (3) based on organ and tissue masses in people with different degrees of adiposity as well as intraindividually before and after weight loss.

## Methods

### Study protocols and subjects

Data analysis involved measures of body composition and REE in individuals who took part in previous studies at the Institut für Humanernährung und Lebensmittelkunde, Christian-Albrechts-University Kiel. Briefly, healthy Caucasian men and women were recruited from the general public. All subjects had a normal physical examination, no use of lipid-lowering, hypoglycaemic or antihypertensive medication, no history of cardiovascular or metabolic disease, and a normal thyroid function. Women were non-pregnant and non-lactating. A total number of 301 men and women with a wide age and BMI range (aged 18–78 years with a BMI between 16.8 and 58.7 kg/m^2^ = study population 1; Tables 2 and 3) were analyzed cross-sectionally including the analysis of organ and tissue masses. In a longitudinal analysis, healthy overweight and obese participants were recruited for a dietary-weight loss intervention trial. In a subsample of 47 men and women organ and tissue masses were analyzed before and after weight loss (study population 2; Table 4). Study population 3 (Table 5; Bosy-Westphal et al., 2013) consisted of 110 women whose body composition before and after weight loss was assessed by densitometry only. Inclusion criteria and recruitment of participants for the dietary intervention trial was described previously (Bosy-Westphal et al., 2009). The weight loss program consisted of weekly individual counseling by a registered dietician and a 13 ± 3 week low-calorie, nutritionally balanced self-selected diet containing 800–1000 kcal/day where of 434 kcal/day were supplied as a very-low-energy-diet (BCM^©^-Diät, PreCon, Darmstadt, Germany, ingestion of two shakes/day provided all nutrients according to RDA, 37.3 g protein, 38.8 g carbohydrate, and 13.5 g fat). The studies were approved by the medical faculty ethics committee of the Christian-Albrechts-University Kiel. All subjects provided their fully informed and written consent before participation.

**Table 2:** **Regression equations for prediction of resting energy expenditure from two different models of body composition analysis (*n* = 301 men and women, BMI 28.4 ± 6.1 kg/m^2^, age 40.9 ± 13.9 years, *study population 1*)**.

**REE, MJ/day =**	***R*^2^**	**SEE**
0.088 × FFM + 0.027 × FM + 1.237	0.80	0.54
0.788 × liver + 1.004 × brain + 0.064 × SM + 0.057 × RM + 0.022 × AT + 1.891 × kidneys – 0.134	0.84	0.49

SM, skeletal muscle; RM, residual mass; AT, adipose tissue.

**Table 3:** **Comparison of basal characteristics and resting energy expenditure adjusted for body composition between normal weight, overweight, and obese participants of *study population 1***.

	**Normal weight *n* = 105**	**Over weight *n* = 92**	**Obese *n* = 104**
Age, years	39.6 ± 15.0	46.2 ± 14.4	37.6 ± 10.7
BMI, kg/m^2^	22.4 ± 1.9	27.4 ± 1.6	37.6 ± 10.7^‡^
FM, %	24.2 ± 9.2	30.5 ± 8.7	43.6 ± 8.2^‡^
FM, kg	15.9 ± 6.1	25.3 ± 6.8	45.3 ± 12.4^‡^
Skeletal muscle mass, kg	24.7 ± 5.8	27.8 ± 6.5	30.9 ± 7.3^‡^
Skeletal muscle mass, kg/FFM, kg	0.49 ± 0.07	0.48 ± 0.11	0.53 ± 0.06^‡^
Organ mass, kg	3.44 ± 0.37	3.86 ± 0.44	4.05 ± 0.49^‡^
Organ mass, kg/ FFM, kg	0.070 ± 0.008	0.067 ± 0.009	0.071 ± 0.009^‡^
REE_measured_, MJ/day	6.08 ± 0.88	7.01 ± 1.00	7.69 ± 1.14^‡^
REE_measured-predicted according to organ and tissue masses_, MJ/day	0.09 ± 0.46	0.09 ± 0.50	0.16 ± 0.61^**^
REE_measured-predicted according to FFM and FM regression in normal weight subjects^#^,_ MJ/day	−0.04 ± 0.44	0.01 ± 0.54	0.35 ± 0.63^***^^‡^
REE_measured-predicted according to FFM and FM regression in obese subjects^##^,_ MJ/day	−0.33 ± 0.45^***^	−0.36 ± 0.52^***^	−0.03 ± 0.62^‡^

** P < 0.01;

*** P < 0.001 difference between measured and predicted REE, paired t-test.

‡ P < 0.05 significantly different from normal and overweight subjects, ANOVA.

# Regression equation: REE, MJ/day = 0.084 × FFM, kg + 0.007 × FM, kg + 1.748, R^2^ = 0.75; SEE = 0.44.

## Regression equation: REE, MJ/day = 0.087 × FFM, kg + 0.015 × FM, kg + 1.963, R^2^ = 0.71; SEE = 0.62.

**Table 4:** **Comparison of body composition and resting energy expenditure between baseline and after weight loss (*n* = 47 males and females, *study population 2* with organ-tissue analysis)**.

	**Before weight loss**	**After weight loss**
Age, years	36.3 ± 6.3	
Weight, kg	105.5 ± 17.9	95.1 ± 16.6^***^
BMI, kg/m^2^	35.6 ± 4.5	32.1 ± 4.3^***^
FFM, kg	58.8 ± 11.4	58.2 ± 10.8
FM, kg	46.8 ± 13.7	36.9 ± 14.5^***^
Skeletal muscle, kg	32.0 ± 8.1	27.5 ± 6.7^***^
Organ mass, kg	4.03 ± 0.50	3.84 ± 0.48^***^
REE, MJ/day	7.73 ± 1.24	7.13 ± 0.96^***^
REE_measured-predicted according to FFM and FM regression before weight loss,_ MJ/day	0.03 ± 0.70	−0.32 ± 0.72^***^
REE_measured-predicted according to organ and tissue masses_, MJ/day	0.19 ± 0.66	0.16 ± 0.55

*** P < 0.001 difference between before and after weight loss, paired t-test.

Organ mass = sum of brain, heart, liver, and kidney masses.

**Table 5:** **Comparison of body composition and resting energy expenditure between baseline and after weight loss (*n* = 110 females, *study population 3* without organ-tissue analysis)**.

	**Before weight loss**	**After weight loss**
Age, years	36.2 ± 9.7	
Weight, kg	107.9 ± 21.8	93.9 ± 17.1^***^
BMI, kg/m^2^	38.0 ± 7.0	33.0 ± 4.9^***^
FFM, kg	54.3 ± 7.4	52.8 ± 6.1^**^
FM, kg	53.6 ± 16.6	41.1 ± 13.3^***^
FM, %	48.8 ± 6.3	42.8 ± 7.1^***^
REE, MJ/day	7.46 ± 1.06	6.79 ± 0.84^***^
REE_measured-predicted according to FFM and FM regression before weight loss_, MJ/day	0.01 ± 0.64	−0.23 ± 0.51^***^
REE_measured-predicted according to %FM-specific FFM-regressions_, MJ/day^†^	− 1.23 ± 0.65	−1.28 ± 0.58

** P < 0.01;

*** P < 0.001 difference between before and after weight loss, paired t-test.

† Equations were published in Bosy-Westphal et al. (2009).

### Anthropometric measurements and body composition analysis

Body weight (±100 g) was measured in underwear on an electronic Tanita scale coupled to the BOD-PODTM system. Height was measured without shoes on a stadiometer (seca, Hamburg Germany) to the nearest 0.5 cm.

### Magnetic resonance imaging

Volumes of internal organs were assessed by MRI (Magnetom Avanto 1.5-T, Siemens Medical Systems, Erlangen, Germany). Subjects were examined in a supine position with their arms extended above their heads. Transversal images were obtained from wrist to ankle by using a contiguous axial T1 weighted gradient-echo sequence (TR 157 ms, TE 4 ms, flip angle 70°, voxel size 3.9 × 2 × 8 mm^3^). Only images from the head, abdominal and thoracic regions were included in the present analysis. The protocol for the brain comprised contiguous 4 mm slices with 1 mm inter-slice gaps (TR 313 ms, TE 14 ms). For the rest of the body, images were obtained with 8 mm slice thickness and 2 mm inter-slice gaps. Image acquisition for volumetric assessment of the thoracic and abdominal region was obtained in breath-hold and heart mass was assessed using a breath navigated and pulse triggered T2-weighted HASTE sequence, (imaging parameters: TR 700 ms, TE 24 ms, flip angle 160°, voxel size 2.2 × 1.3 × 8 mm, turbo factor 106). All images were segmented manually (Slice-O-Matic, Tomovision 4.3 Software, Montreal, Canada). Total organ volume was determined from the sum of all areas (cm^2^) multiplied by slice thickness. Volume data were transformed into organ mass using the following densities: 1.036 g/cm^3^ for brain, 1.06 g/cm^3^ for heart and liver and 1.05 g/cm^3^ for kidneys (Elia, 1992).

### Densitometry

Air-displacement plethysmography was performed using the BOD-PODTM device (Life Measurement Instruments., Concord, CA, USA). Subjects were measured in tight fitting underwear and a swimming cap. Two repeated measurements of body volume were performed and averaged. Measured thoracic lung volume was subtracted from body volume. BOD-POD® software was used to calculate body density as body weight divided by body volume and FM% using Siri's equation (Siri, 1993). Fat free mass (FFM, kg) was calculated as: weight (kg)—FM (kg). The coefficient of variation for repeated measurements of %FM was 2.4%.

### Resting energy expenditure

Indirect calorimetry was performed in the morning between 7.30 and 9.00 a.m. after an overnight fast (ventilated hood system: Vmax Spectra 29 n; SensorMedics BV, Bilthoven, Netherlands; software Vmax, version 12-1A). The minimum duration of measurement was 35 min and the first 10 min were discarded. Flow calibration was performed by a 3L-syringe and gas analyzers were calibrated before and every 5 min during the run. Data were collected every 20 s and acquired VO2 and VCO2 were converted to REE (kcal/24 h) using the abbreviated equation of Weir. The CVs for repeated REE-measurements were 5.2%.

REE was normalized for FFM and FM by regression analysis (REEadjusted FFM + FM) and also accounting for detailed body composition by subtracting REE calculated from organ and tissue masses (REEc) from measured REE (REEmeasured-calculated). Calculation of REE was based on the sum of eight body compartments (brain, heart, liver, kidneys, skeletal muscle mass, bone mass, adipose tissue, and residual mass) times the corresponding tissue-respiration rate, using the specific tissue-metabolic rates as reported by Elia (1992; see Müller et al., 2002). Residual mass was calculated as body mass minus the sum of brain, heart, liver, kidneys, skeletal muscle mass, and adipose tissue. The metabolic activity of residual mass was assumed to be 30 kJ/kg/day (Bosy-Westphal et al., 2009).

REEc (kJ/day) = (1008 × brain mass) + (840 × liver mass) + (1848 × heart mass) + (1848 × kidney mass) + (55 × skeletal muscle mass) + (19 × adipose tissue) + (30 × residual mass).

Adipose tissue was calculated from FM assuming a fat content of 90%.

Skeletal muscle mass was derived from appendicular lean soft tissue measured by DXA (Hologic Discovery A densitometer, Hologic, Inc., Bedford, Massachusetts, USA) using equations validated against whole body MRI (Kim et al., 2002).

### Statistics

Data are expressed as means ±SD. Comparisons between independent groups were analyzed by ANOVA using Bonferroni *post-hoc* test for comparisons between three BMI groups. Intraindividual comparisons between baseline values and after weight loss were analyzed using paired samples *t*-test. Relationships between variables were sought by correlation analysis (Pearson's r). Two-tailed *P*-values <0.05 were considered to indicate statistical significance. Data analyses were performed with SPSS statistical software (SPSS 15.0, Inc., Chicago, USA).

## Results

### Impact of different ways to normalize REE in normal and overweight subjects

The results of stepwise regression analyses predicting REE from two different models of body composition analysis are given in Table 2 for *study population 1*. The coefficient of determination is only marginally lower and standard error of estimate is slightly higher for the prediction model based on absolute values of FFM and FM when compared with a prediction model based on organ and tissue masses. Both models were not significantly improved by the inclusion of gender, age, or %FM.

Comparing the different models for adjusting REE between normal weight, overweight and obese participants reveals that REE predicted from organ and tissue masses does not fully explain the higher REE in obese subjects whereas the difference between measured and predicted REE was not significant in normal and overweight subjects (Table 3). However, there was no significant difference in REE_measured−calculated by organ and tissue masses_ when comparing obese vs. lean/overweight participants. By contrast, the difference in REE between normal-/overweight and obese participants was significant after adjusting REE using regression analysis based on FFM and FM. Transferring the regression equation derived in obese participants to normal-/overweight subjects' leads to a seemingly lowered metabolic rate in these groups. Conversely, an equation derived in normal weight participants and applied to normalize REE in obesity leads to the result of an elevated metabolic rate in obese subjects.

Table 4 shows the results for REE adjusted for body composition before and after weight loss in *study population 2* (with detailed analysis of organ and tissue masses). Mean weight loss was −10.4 ± 4.2 kg (*p* < 0.001) and mainly consisted of FM. Lean mass was preserved and did not significantly differ between baseline and after weight loss. REE adjusted for FFM and FM was based on a regression equation developed at baseline and showed a significant drop in metabolic rate after weight loss (*p* < 0.001) whereas REE adjusted for changes in organ and tissue masses did not change with follow-up.

In *study population 3* (see Table 5), body composition analysis was performed by a 2-compartment model only. Similar to *study population 2*, weight loss mainly consisted of FM and adjusting REE for FFM and FM based on regression analysis performed at baseline led to a lower adjusted REE after weight loss. In this group of women we also compared REE measured by indirect calorimetry to REE predicted from %FM-specific REE-prediction equations (based on FFM and FM) that were derived in a large female database and have been previously published by our group (Bosy-Westphal et al., 2009). The comparison between adjusted REEs before and after weight loss showed no significant differences.

Using data from *study population 1*, we analyzed the contribution of different organ masses to FFM with increasing adiposity (Table 6). Surprisingly, the contribution of liver, heart (women only), kidney, and skeletal muscle masses per kg FFM increased with increasing %FM in both genders but the ratios of brain/FFM (women only) and residual mass/FFM that showed an inverse association with %FM. Accordingly, regression analysis using REE_calculated from organ and tissue masses_ (MJ)/FFM (kg) as the dependent variable and %FM as the independent variable revealed a significant positive relationship (REE_calculated from organ and tissue masses_ /FFM = 0.001 × %FM + 0.105; *R*^2^ = 0.44).

**Table 6:** **Coefficients of correlation between organ and tissue masses/FFM and adiposity (%FM) stratified by gender in *study population 1***.

	**%FM vs.**	
	**Women (*n* = 179)**	**Men (*n* = 122)**
Skeletal muscle (kg)/FFM (kg)	0.30^***^	0.27^**^
Residual mass (kg)/FFM (kg)	−0.57^***^	−0.44^***^
Brain mass (kg)/FFM (kg)	−0.25^**^	−0.05
Liver mass (kg)/FFM (kg)	0.39^***^	0.53^***^
Heart mass (kg)/FFM (kg)	0.17^*^	−0.18
Kidney masses (kg)/FFM (kg)	0.38^***^	0.21^*^

* P<0.05;

** P < 0.01;

*** P < 0.001.

## Discussion

### REE normalization in lean and obese people and before and after weight loss

Our results show that an “REE vs. FFM and FM equation” that (1) was derived in obese participants and applied to lean people or (2) was derived before weight loss and applied to data after weight loss leads to the erroneous conclusion of a lower metabolic rate (i) in lean persons (Table 3) and (ii) after weight loss (Table 4). By contrast, the normalization of REE for organ and tissue masses (REEmeasured—REE calculated from organ and tissue masses) was not significantly different between lean and obese or between baseline and after weight loss.

However, the measurement of organ and tissue masses to normalize REE is not suitable in daily clinical practice and has only been used in a limited number of studies. Only recently, a substantial drop in specific REE (−5.4 ± 1.1 kcal/day) has been deduced after diet and exercise induced massive weight loss (−57.6 kg) despite a relative preservation of FFM (Johannsen et al., 2012). This diagnosis was based on a regression analysis that predicted REE from baseline levels of FFM, FM, age, and sex and was applied to FFM and FM measured after weight loss. The authors concluded that the high difference between measured and predicted REE after weight loss corresponds to a dramatic metabolic slowing that cannot be explained by changes in body composition. However, it is possible that in this study “metabolic slowing” did not occur *despite* the preservation of lean mass but as a direct consequence of it. The preferential loss in FM (−47.1 kg, 83 ± 8% of weight loss) likely contributed to an overestimation of adaptive thermogenesis because the regression analysis from baseline values was valid for a mean %FM of 49 ± 5%. After weight loss, participants had a mean %FM of 28 ± 10% only. In a previous publication we have shown that the regression coefficients for FFM and FM differ between different degrees of adiposity (Bosy-Westphal et al., 2009). Therefore, a regression analysis derived from participants with a higher %FM cannot be used to normalize REE in a leaner group of people without bias. In line with this argumentation, the application of our previously published %FM-specific regression equations (Bosy-Westphal et al., 2009) to normalize REE before and after weight loss in the article of Johannsen et al. (2012) leads to a comparable accuracy of REE prediction at both time points (REEmeasured—predicted at baseline + 120 kcal/day and after weight loss + 112 kcal/day). The small inaccuracy of REE prediction at both time points is likely due to differences in body composition analysis (DXA vs. BIA) and the fact that our equations apply to women only. Using these equations to normalize REE also eliminated the difference in REE before and after weight loss (Table 5).

A physiologic explanation of the seemingly higher metabolic rate before weight loss when adipocytes are large and filled with lipids compared to the smaller fat cells after weight loss induced lipolysis remains unclear. It is tempting to speculate that obesity associated co-morbidities (Bosy-Westphal et al., 2008) as well as the endocrine function of adipose tissue (Kaiyala and Schwartz, 2011) contribute to an elevation of REE that is normalized after weight loss. However, the fact that REE normalized for organ and tissue masses can be applied to lean and obese people with sufficient accuracy (Table 3, Bosy-Westphal et al., 2004; Wang et al., 2012) indicates that changes in the regression coefficients of FFM and FM with increasing adiposity may be partly explained by changes in the composition of lean mass (i.e., the ratio of high to low metabolic organs). In line with this observation, metabolic activity of FFM (e.g., REE predicted from organ and tissue masses/FFM) increased with increasing %FM. This may be due to increased masses of liver, heart and kidneys per kg FFM with increasing %FM whereas brain mass per FFM and low metabolically active residual mass/FFM decreased (Table 6).

### Normalization of REE based on organ and tissue masses

The concept of relating metabolic rate to organ size was introduced by Holliday et al. as early as in 1967. Brain, heart, liver, and kidneys comprise only 5–6% of body weight, but contribute to >80% of REE, whereas other components such as muscle, adipose tissue or bone mass have low specific resting metabolic rates (Smith and Hoyer, 1962; Grande, 1980; Elia, 1992). However, initial studies published between 1992 and 1997 did not find a significant contribution of tissue masses to the REE variance beyond that explained in FFM (Deriaz et al., 1992; McNeill et al., 1995; Sparti et al., 1997). This may be due to the small numbers of subjects investigated, low inter-individual differences in body composition, and methodological limitations that did not allow a differentiated volumetric assessment of all organs and tissues.

Today, functional body composition analysis at the organ and tissue level adds to our understanding of inter-individual variance in REE (Gallagher et al., 2000, 2006; Illner et al., 2000; Hsu et al., 2003; Midorikawa et al., 2007; for a review see Müller et al., 2002, 2009). Differences in organ and tissue mass contribute to differences in REE between underweight, overweight and obese subjects (cf. Bosy-Westphal et al., 2004), Sumo wrestlers and untrained college students (Midorikawa et al., 2007) as well as between African American and white adults (Gallagher et al., 2006). Organ and tissue modeling of REE has also shown that the specific metabolic rates apply equally well in both genders (i.e., differences in REE normalized for FFM between men and women are explained by sex-differences in FFM-composition, Wang et al., 2011). A lower mass of high metabolic rate organs also contributes to the lower REE in elderly individuals (Bosy-Westphal et al., 2003) but did not fully account for the differences in REE observed between young and elderly people (Gallagher et al., 2000). This is also confirmed in a recent analysis performed in a greater study population (comprising 131 adults aged 21–73 years with a BMI < 30 kg/m^2^) which showed that the specific metabolic rates of major organs (brain, heart, liver, and kidneys) published by Elia (1992) are valid in the younger age groups but were estimated to be 3% lower in the group >50 years (Wang et al., 2010).

In addition, a greater mass of high metabolic rate organs does not fully explain the higher metabolic rate observed in children (Hsu et al., 2003). This is likely explained by the metabolic costs of growth (Holliday, 1971).

Inter-species comparison shows that specific organ metabolic rate varies with body mass, with higher energy expenditure per unit organ mass in smaller mammals (Couture and Hulbert, 1995). There is, however, no evidence for a mass dependency of specific organ metabolic rate in humans with a range of body mass from 44 to 104 kg and a normal FM (Later et al., 2008).

### Proposal of a new way of REE normalization including information on %FM

REE normalization based on organ and tissue masses is expensive, time-consuming and methodically complex that is not without limitation (e.g., assumption about the lipid content of adipose tissue and liver) and confined to smaller sample sizes. Normalization of REE based on FFM and FM therefore remains indispensable in daily practice and the majority of scientific studies. We propose the use of %FM-specific regression equations for REE vs. FFM and FM (Bosy-Westphal et al., 2009). The advantage of this method is a plausible approach that provides new insights on the contribution of adiposity to metabolic rate. The information on adiposity is an advantage over the commonly applied normalization of REE using FFM and FM in kg only. FM in kg does not reflect adiposity of the body because a large and heavy person and a smaller person can have the same amount of FM but differ greatly in their adiposity (%FM). Thus, the influence of adiposity on the composition of lean mass (Table 6) or the impact of obesity on co-morbidities and the endocrine function of adipose tissue can only be taken into account by REE normalization that includes information on adiposity. This argument is supported by our finding, that %FM-specific regression of REE vs. FFM and FM (derived from Bosy-Westphal et al., 2009) revealed no difference in metabolic rate before and after weight loss whereas the conventional approach using only absolute values of FFM and FM for normalization leads to a significant difference between measured and predicted REE.

### Study limitations

FFM was measured by air-displacement plethysmography (densitometry, Table 3) that is known to overestimate the loss in FM (and underestimate the loss in lean mass) during weight loss due to similar densities of fat and water. This bias may have contributed to an overestimation of lean mass after weight loss that could explain the significantly lower REE adjusted for FFM and FM. In addition, some authors found that the hydration of FFM did not normalize after weight loss (Das et al., 2003). The higher hydration of FFM in obese and weight reduced subjects may thus contribute to an overestimation of FFM. Therefore, the lack of all two compartment methods to accurately assess changes in body composition with weight loss may mimic a reduction in specific metabolic rate.

The finding of a seemingly higher metabolic activity of FFM with increasing adiposity (due to an increase in organ mass/FFM) was unexpected and may have been overestimated by an increased liver fat content with increasing adiposity. Future studies should investigate the contribution of liver fat to the specific metabolic rate of this tissue.

In contrast to the present findings, in a previous publication conducted in overweight and obese women who lost weight in response to a low-calorie diet for 3 months we found that about 40% of the decline in REE were not explained by a decrease in organ and tissue masses and were thus attributed to adaptive thermogenesis (Bosy-Westphal et al., 2009). The reason for the discrepant findings remains unclear. However, the population of the present manuscript differs from our former publication and also included men. Organ mass and skeletal muscle mass were both higher in the present population. In addition, the loss in skeletal muscle mass was also higher. Because appendicular lean soft tissue measured by DXA was used to calculate skeletal muscle mass, and these equations differ for men and women, the relative maintenance of muscle mass in the former publication could have been overestimated thus leading to a reduction in REE adjusted for organ and tissue masses. These methodological limitations in addition to uncertainties about organ lipid content and their impact on organ specific metabolic rate as well as organ hydration in response to weight loss add to uncertainty about the ‘quantification' of adaptive thermogenesis in human weight regulation, which is therefore likely to remain more of a concept than a measurable entity (Dulloo et al., 2012; Müller and Bosy-Westphal, 2012).

Finally, our %FM-specific REE normalization approach (Bosy-Westphal et al., 2009) comprises only women because a large number of individuals in the same %FM-category differing widely in FFM is required to derive %FM-specific equations predicting REE from FFM. However, having five different equations for REE-normalization depending on %FM has some drawbacks due to abrupt changes depending on the cut-off. Future studies should develop a more continuous way to adjust REE for FFM and FM.

The prediction of skeletal muscle mass from lean soft tissue of the extremities measured by DXA cannot account for the higher content of connective tissue with increasing adiposity (Schautz et al., 2012) and may therefore overestimate skeletal muscle mass in obesity. In addition, the assumption of a constant density (i.e., lipid content) of adipose tissue and liver mass is likely violated in obesity and contributes to a bias when normalizing REE for organ and tissue masses in obese patients.

The impact of adiposity associated co-morbidities (SNS-activity, insulin resistance, blood pressure) or endocrine function (fT3, leptin etc.) was analyzed in our previous publications (Bosy-Westphal et al., 2008) and is beyond the scope of the present paper.

In summary, the normalization of REE for body composition is not trivial when comparing people with a wide range of adiposity as well as before and after substantial weight loss. The most established method for normalization of REE based on FFM and FM may lead to spurious conclusions about metabolic rate in obesity and the phenomenon of weight loss-associated adaptive thermogenesis. Organ-tissue based models are superior to equations based on FFM and FM. However, information on organ and tissue mass is rarely available and using %FM in addition to FFM and FM for adjusting REE may account for the increase in specific metabolic rate of lean mass with increasing adiposity and thus provide new insights into the old controversy about the impact of specific REE on the cause and consequence of obesity.

## Funding

The study was funded by a grant of the German Ministry of Education and Research (BMBF 0315681), BMBF Competent Network of Obesity (CNO) and the German Research Foundation (DFG Bo 3296/1-1 and DFG Mü 714/ 8-3).

### Conflict of interest statement

The authors declare that the research was conducted in the absence of any commercial or financial relationships that could be construed as a potential conflict of interest.
